# Bone marrow harvesting from paediatric patients undergoing haematopoietic stem cell gene therapy

**DOI:** 10.1038/s41409-019-0573-6

**Published:** 2019-05-31

**Authors:** Francesca Tucci, Marta Frittoli, Federica Barzaghi, Valeria Calbi, Maddalena Migliavacca, Francesca Ferrua, Francesca Fumagalli, Laura Lorioli, Laura Castagnaro, Marcella Facchini, Claudia Fossati, Stefano Zancan, Paola Massariello, Michele Manfredini, Giulia Consiglieri, Daniele Canarutto, Salvatore Recupero, Francesco Calzatini, Miriam Casiraghi, Silvia Darin, Gigliola Antonioli, Roberto Miniero, Rossana Fiori, Paolo Silvani, Matilde Zambelli, Sarah Marktel, Salvatore Gattillo, Raffaella Milani, Luca Santoleri, Fabio Ciceri, Alessandra Biffi, Maria Pia Cicalese, Maria Ester Bernardo, Alessandro Aiuti

**Affiliations:** 1 Pediatric Immunohematology Unit and BMT Program, IRCCS San Raffaele Scientific Institute, Milan, Italy; 2 Tor Vergata University, Rome, Italy; 3 San Raffaele Telethon Institute for Gene Therapy (SR-Tiget), IRCCS San Raffaele Scientific Institute, Milan, Italy; 4 Vita-Salute San Raffaele University, Milan, Italy; 5 Neurology Department, IRCCS San Raffaele Scientific Institute, Milan, Italy; 6 Molmed S.p.A, Milan, Italy; 7 Department of Pediatrics, Pugliese-Ciaccio Hospital Magna Graecia University, Catanzaro, Italy; 8 Department of Anesthesia and Critical Care, IRCCS San Raffaele Scientific Institute, Milan, Italy; 9 Immunohematology and Transfusion Medicine Unit, IRCCS San Raffaele Scientific Institute, Milan, Italy; 10 Hematology and Bone Marrow Transplantation Unit, IRCCS San Raffaele Scientific Institute, Milan, Italy; 11 Gene Therapy Program, Dana-Farber/Boston Children’s Cancer and Blood Disorders Center and Harvard Medical School, Boston, MA 02115 USA

**Keywords:** Stem-cell research, Medical research

## Abstract

Collection of an adequate amount of autologous haematopoietic stem progenitor cells (HSPC) is required for ex vivo manipulation and successful engraftment for certain inherited disorders. Fifty-seven paediatric patients (age 0.5–11.4 years) underwent a bone marrow harvest for the purpose of HSPC gene therapy (GT), including adenosine deaminase-severe combined immunodeficiency (ADA-SCID), Wiskott–Aldrich syndrome (WAS) and metachromatic leukodystrophy (MLD) patients. Total nucleated cells and the percentage and absolute counts of CD34+ cells were calculated at defined steps of the procedure (harvest, CD34+ cell purification, transduction with the gene transfer vector and infusion of the medicinal product). A minimum CD34+ cell dose for infusion was 2 × 10^6^/kg, with an optimal target at 5–10 × 10^6^/kg. Median volume of bone marrow harvested was 34.2 ml/kg (range 14.2–56.6). The number of CD34+ cells collected correlated inversely with weight and age in all patients and particularly in the MLD children group. All patients reached the minimum target dose for infusion: median dose of CD34+ cells/kg infused was 10.3 × 10^6^/kg (3.7–25.9), with no difference among the three groups. Bone marrow harvest of volumes > 30 ml/kg in infants and children with ADA-SCID, WAS and MLD is well tolerated and allows obtaining an adequate dose of a medicinal product for HSPC-GT.

## Introduction

Gene therapy (GT) with autologous haematopoietic stem/progenitor cells (HSPC) is a promising treatment for primary immunodeficiency, such as adenosine deaminase-severe combined immunodeficiency (ADA-SCID) [1–4] and Wiskott–Aldrich syndrome (WAS) [5, 6], and inherited metabolic disorders such as metachromatic leukodystrophy (MLD) [7, 8].

Collection of high numbers of CD34+ HSPC is therefore crucial for in vivo administration of adequate numbers of gene-modified HSPC.

Bone marrow (BM) harvest from iliac crests represents a suitable modality of stem cell procurement in healthy paediatric subjects donating for allogeneic haematopoietic stem cell transplantations (HSCT) of their siblings [9, 10], as well as for autologous HSPC transplantations [11, 12], autologous backup [13] or, more recently, for GT. Indeed, it has been used for all our San Raffaele-Telethon Institute for Gene Therapy (SR-TIGET) trials for ADA-SCID, WAS and MLD [1, 4, 5, 7], as well as in other gene therapy clinical trials for primary immune deficiencies [2, 3, 6, 14, 15].

A direct correlation between cell-dose and engraftment level is a well-known observation in haematopoietic transplant medicine with a recommended minimal dose of > 4 × 10^8^ total nucleated cells (TNC) [16] or > 3 × 10^6^ CD34+ cells/kg of body weight [17] for allogeneic transplantation. For GT clinical trials, usually a minimum target dose at infusion of 2 × 10^6^ CD34+ cells, with a target of 5–10 × 10^6^ CD34+ cells/kg, is recommended. Data from the ADA-SCID GT clinical trial suggest that the engraftment of transduced progenitors is dependent on the dose of CD34+ cells administered [15]. In WAS patients, the degree of myeloid cell engraftment and of platelet reconstitution correlated with the dose of gene-corrected cells administered [6].

GT requires collecting a higher number of cells than that usually recommended for a conventional unmanipulated transplantation, due to the expected cell loss occurring during subsequent procedures, such as mononuclear cell purification, CD34+ cell positive selection and ex vivo cell culture with transduction and possibly cryopreservation. Therefore, we aimed at collecting at least 5–8 × 10^6^/kg before any manipulation to achieve the minimal target dose.

Here, we evaluated the BM yield, mainly in terms of tolerability and content of HSPCs harvested in children affected by ADA-SCID, WAS and MLD undergoing GT. We also evaluated the presence of patient and harvest-related features that could influence the autologous HSPC collection.

## Patients and methods

### Patients’ and procedure description

We analysed 57 infants and children (age 0.5–11.4 years) who underwent from October 2002 to November 2017 a BM harvest for the purpose of HSPC-GT, including 23 ADA-SCID, 6 WAS and 28 MLD patients. Details about patients’ enrolment are reported as supplementary data. Written informed consent was obtained from the donors’ parents or legal guardians in accordance with Italian law.

According to the respective clinical protocol/guideline, a HSPC backup was harvested and cryopreserved unmanipulated to be used in case of poor engraftment or technical issues with product manufacture. About 1 month after back-up harvest, patients underwent a main harvest for the preparation of a transduced medicinal product. Patients’ CD34+ cell manipulation and transduction were performed at MolMed S.p.A., a certified Good Manufacturing Practice (GMP) facility.

On average, BM volume collected was set at 20–30 ml/kg of donor weight according to the clinical trial protocols.

To achieve an optimal dose of 5–10 × 10^6^ CD34+ transduced cells/kg at infusion, our target of CD34+ cell dose collected was 10–20 × 10^6^ CD34+ cells/kg before any manipulation. Initial, mid- and final harvest TNC count was used to set the total volume to be harvested. Percentage and absolute counts of CD34+ cells were calculated by cell count and cytofluorimetric analysis measured during the procedure at defined steps of the procedure: (1) at the end of BM harvest; (2) CD34+ cell purification; (3) positive selection; (4) transfer vector; (5) quality controls and (6) infusion of the medicinal product.

### Statistical analysis

Statistical analysis was performed with Prism version 6 (GraphPad Software, San Diego, CA, USA). For continuous variables, the results are expressed as medians and ranges. Comparison among patients’ harvest parameters and biological characteristics was performed by linear regression analysis or by a two-tailed Student's t test. Comparison among three or more groups was performed by ANOVA with Bonferroni correction. A *p*-value <0.05 was considered statistically significant. If a *p*-value is ≤0.05, ≤ 0.01, ≤ 0.001 or ≤ 0.0001, graphs are flagged with one (*), two (**), three (***) or four stars (****), respectively.

## Results

We analysed 57 paediatric patients who underwent BM harvest at our institution for the purpose of HSPC collection for GT (refs. [4, 5, 8] and data not shown). Data from 23 ADA-SCID patients (16 males, 7 females), 6 male WAS patients and 28 MLD patients (15 males, 13 females) are summarised in Table 1. In the MLD group, 16 were affected by late-infantile (LI) variant and 12 by early-juvenile (EJ) variant.

**Table 1:** Summary data of bone marrow harvests for GT

Disease	Age at GT (years)	Weight (kg)	Harvested volume (ml)	Volume/kg	Collected TNC (×10^8^)/kg	CD34+ (%)	Collected CD34+ cells (×10^6^)/kg	Selected CD34+ cells (×10^6^)/kg	Infused CD34+ cells (×10^6^)/kg
ADA-SCID (*n* = 23)	1.7 (0.5–6.0)	10.1 (5.9–26.5)	303.0 (190.0–1167.5)	31.2 (14.2–48.6)	4.2 (2.9–6.8)	5.7 (2.0–6.7)^a^	28.9 (9.8–43.1)^a^	13.8 (4.4–22.9)	10.7 (3.7–19.7)
WAS (*n* = 6)	2.1 (0.9–6.0)	12.7 (8.0–26.3)	424.3 (275.0–773.0)	34.2 (29.4–36.5)	5.7 (3.5–6.6)	2.9 (1.8–5.2)	15.8 (10.8–18.7)	9.1 (4.5–14.5)	9.0 (3.7–14.1)
MLD (*n* = 28)	1.3 (0.5–11.4)	11 (7.3–25.3)	420.0 (194.0–1132.0)	36.9 (17.6–56.6)	6.3 (3.4–9.4)	3.0 (1.0–7.7)	16.3 (5.6–65.9)	9.6 (3.9–34.7)	10.3 (3.8–25.9)
Total (*n* = 57)	1.5 (0.5–11.4)	10.6 (5.9–26.5)	348.0 (190.0–1167.5)	34.2 (14.2–56.6)	5.1 (2.9–9.4)	3.1 (1.0–7.7)	17.3 (5.6–65.9)	10.4 (3.9–34.7)	10.3 (3.7–25.9)

Data indicate median and range

^a^Data available for the last consecutive eight ADA-SCID patients

All patients underwent a back-up harvest before the main harvest for GT; 54/57 were collected from BM (median 23.5 days of interval between the two procedures) and 3 from mobilised peripheral blood (MPB). Data from back-up harvests are summarised in Suppl. Table 1.

For the main harvest, a median volume of 303.0, 424.3 and 420.0 ml (with an anticoagulant citrate dextrose), corresponding to a median of 31.2, 34.2 and 36.9 ml/kg of body weight, was collected in ADA-SCID, WAS and MLD patients, respectively (Table 1). There were no significant differences among the volumes collected.

**Fig. 1:**
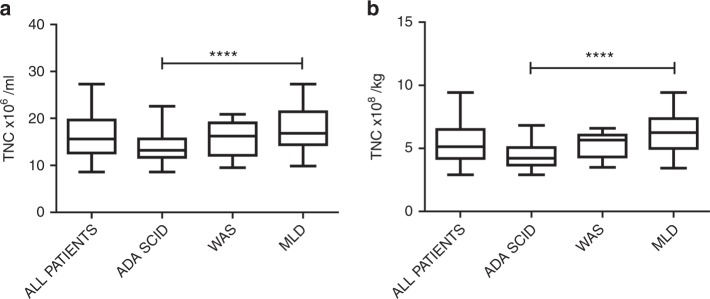
TNC concentration (**a**) and TNC ×10^8^/kg (**b**) in ADA-SCID, WAS and MLD. Each box plot displays the distribution of data based on the five-number summary: minimum, first quartile, median, third quartile and maximum

The harvested BM processed at the GMP facility (MolMed) contained a median of 43.3, 55.5 and 77.7 × 10^8^ TNCs, corresponding to 4.2, 5.7 and 6.3 × 10^8^ cells/kg (Table 1), respectively, in ADA-SCID, WAS and MLD patients. ADA-SCID patients showed a significantly lower TNC concentration (Fig. 1a, ADA-SCID vs MLD patients, *p* < 0.0001) and TNC count/kg (Fig. 1b, ADA-SCID vs MLD patients, *p* < 0.0001).

**Fig. 2:**
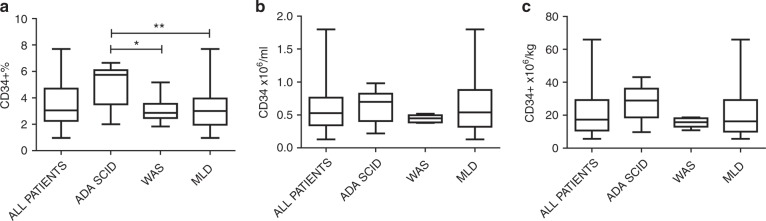
CD34+ cell values are expressed as percentages (**a**), concentration (**b**) and per kg of body weight (**c**) in 8 ADA-SCID, 5 WAS and 28 MLD patients. Data measured after collection at the GMP facility are shown

Evaluation of BM CD34+ cell content was first performed in the operating room in order to guide the collection towards the achievement of the HSPC. CD34+ cell frequency and absolute counts were measured during the procedure and after collection. In the harvested bag, ADA-SCID patients had a higher statistically significant amount of CD34+ percentage of cells than WAS and MLD patients (median CD34+ 5.7, 2.9 and 3.0% in ADA-SCID, WAS and MLD; ADA-SCID vs WAS *p* = 0.033; ADA-SCID vs MLD *p* = 0.0086) (Fig. 2a). In contrast, we did not observe differences among the three groups after normalising for the volume collected and body weight in terms of CD34+ ×10^6^/ml (one-way ANOVA test *p* = 0.48) (Fig. 2b) and CD34+ ×10^6^ cells/kg (one-way ANOVA test *p* = 0.17) (Fig. 2c).

Next, we analysed the influence of patient-related factors with respect to TNC and CD34+ cell harvest. MLD LI patients showed a higher percentage and concentration compared with the EJ variant (median CD34+ ×10^6^/ml in LI 0.76, in EJ 0.33; *p* = 0.0005).

In order to define factors affecting cell dose, the correlation between the collected HSPCs and weight and age was studied.

**Fig. 3:**
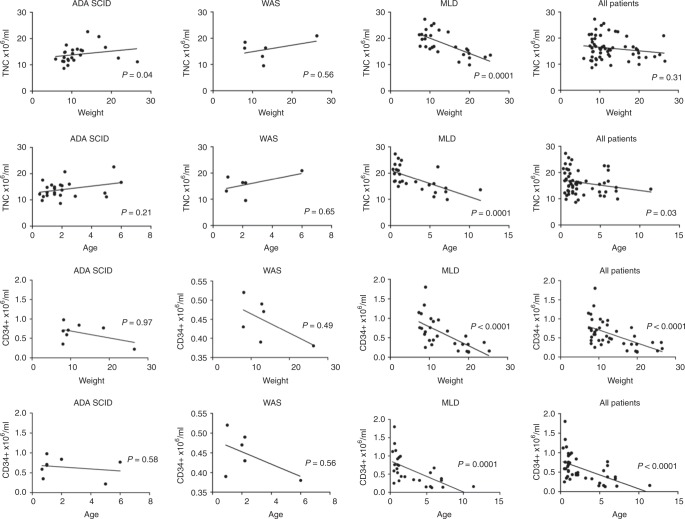
Relationship between the weight (**a**) and age (**b**) of the donor and concentration of TNCs harvested in the three patients subgroups and in all the patients. Relationship between patients’ weight (**c**) and age (**d**) with concentration of CD34+ cells in the BM harvest

Collected TNCs were analysed in relationship with patient’s characteristics, age and weight (Fig. 3a, b). The cumulative group of all patients showed an inverse correlation only for age (*p* = 0.03). MLD patients showed a significant inverse correlation with respect to the two parameters (*p* = 0.0001 for both), indicating a reduction in TNCs harvested for patients with higher weight and age. On the contrary, a direct correlation was seen for ADA-SCID patients, regarding weight (*p* = 0.04) (Fig. 3a) but not age (Fig. 3b). No correlation was seen in the WAS disease setting. Then we investigated the relationship between the CD34+ cell concentration and donor’s weight and age in the three populations. An inverse correlation was documented in the group of all patients and in the subgroup of MLD patients for weight (*p* < 0.0001) (Fig. 3c) and age (*p* = 0.0001) (Fig. 3d), with a similar trend in WAS patients.

**Fig. 4:**
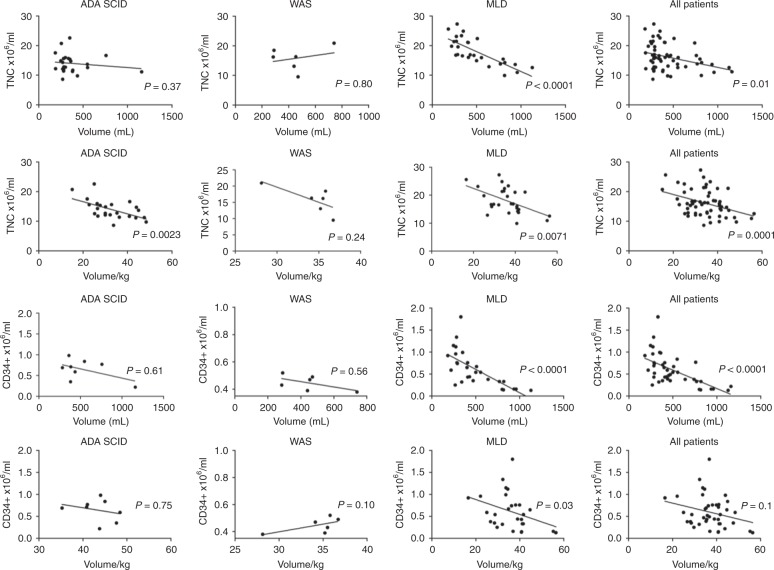
Correlation between TNC concentration and volume (**a**) or volume/kg (**b**) and between CD34+ cell concentration and volume (**c**) or volume/kg (**d**) in the three patients subgroups and in all the patients

The harvested volume was used as a correlation parameter for TNCs and CD34+ cells. In the cumulative patient cohort, TNC and CD34+ cell concentration was shown to be inversely correlated to the harvested volume (*p* = 0.01 and *p* < 0.0001, respectively) (Fig. 4a, c). We hypothesised that in children, where aspiration surface is limited, a possible hemodilution might occur during the harvest procedure. Therefore, volume was normalised to patient’s body weight. We confirmed a significant inverse correlation also for TNC concentration with volume/kg (Fig. 4b) and a trend for CD34+ cell concentration (Fig. 4d), suggesting that increasing the aspirated volume can result in reduced cell concentration. When analysing these parameters by disease, MLD patients displayed a similar pattern for both TNC and CD34+ cells, whereas the correlation was significant for TNC concentration and normalised blood volume in ADA-SCID patients. There was no correlation in WAS patients, possibly also due to the limited sample size of this group.

**Fig. 5:**
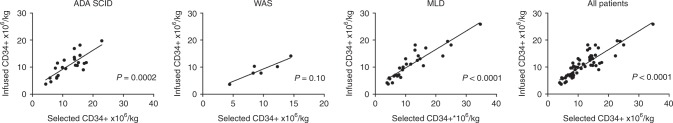
Relationship between selected and infused CD34+ cells in the three patients subgroups and in all the patients

CD34+ cell counts after manipulation and transduction are summarised in Table 1. A median of 10.4 selected CD34+ cells ×10^6^/kg were obtained after HSPC purification; 13.8, 9.1 and 9.6 CD34+ ×10^6^/kg cells were obtained in ADA-SCID, WAS and MLD children, respectively. After transduction, a median of 10.3 CD34+ ×10^6^/kg were infused; 10.7, 9.0 and 10.3 CD34+ cells/kg were infused in the three patients’ groups, in line with the target range (5–10 × 10^6^/kg) of the clinical protocols. No statistical difference was observed within the three disease groups (one-way ANOVA test *p* = 0.71). The infused dose appeared to be directly associated with the amount of CD34+ cells collected and purified after the BM harvest (Fig. 5).

**Fig. 6:**
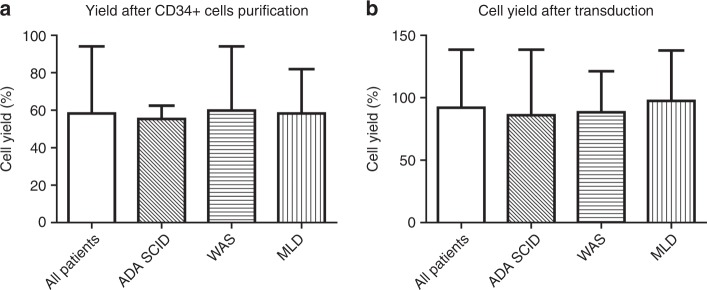
Cell yield during the consecutive steps of the production process. We calculated cell yield between harvest and selection (**a**) and cell yield after the transduction step (**b**)

We next evaluated the cell yield during the different steps of the production process. The median cell yield after CD34+ cell purification was 58.3% (39.5–94.1) and was similar for all the three groups (Fig. 6a). Median cell recovery after transduction was 92.1% (53.6–138.5) with no substantial differences in the three groups, despite different culture conditions among the ADA-SCID, WAS and MLD protocols (Fig. 6b).

**Fig. 7:**
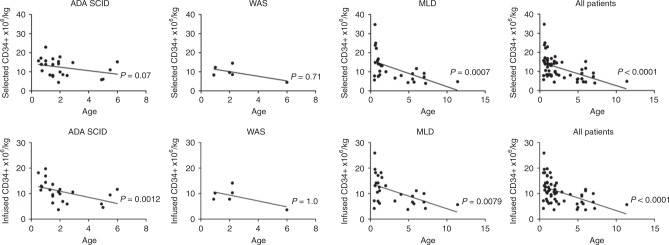
Relationship between age of the donor and CD34+ cells (as CD34+ ×10^6^/kg) after purification (**a**) and after cell manipulation and transduction (**b**)

We also investigated if donor age has a relationship with selected (Fig. 7a) and infused transduced CD34+ cell counts (Fig. 7b). Overall, we observed the same trend previously seen for the unmanipulated HSPCs, with a significant inverse correlation with respect to age (*p* < 0.0001 both for selected and for infused cells). Among the three groups of disease, only selected CD34+ cell/kg of MLD patients showed a reduction of CD34+ cell counts in older patients (*p* = 0.0007) (Fig. 7a). With regard to the infused cell, an indirect correlation with age was seen both in ADA-SCID and in MLD patients (ADA-SCID (*p* = 0.0012; MLD *p* = 0.0079) (Fig. 7b).

## Discussion

The amount of harvested HSPCs is a critical parameter for GT, because it represents the starting material for the preparation of the medicinal product. Harvested CD34+ cells need to be purified and ex vivo cultured to produce the drug product that is infused in the patient. BM historically has been the preferred source for HSPC collection in the GT setting, although MPB has been increasingly adopted in the last few years [9]. To date, few data are available regarding BM harvest in the paediatric population, mainly limited to the maximum amount that can be safely collected and the purification yields of CD34+ cells [18, 19]. Moreover, scattered data on specific diseases, such as primary immunodeficiency or lysosomal storage disorders, are reported [13, 20].

Here, we performed a comprehensive analysis of the tolerability of large-volume collection and of the quality of BM harvest in the context of an autologous GT setting. Moreover, we evaluated the impact of patients’ and harvest-related features on the amount of HSPCs available for cell manipulation and transduction in children enrolled in clinical trials of GT for ADA-SCID, WAS and MLD.

We found that the number of TNCs and HSPCs harvested and selected for GT, depends on patient- and harvest-related factors. In our study, we aimed at infusing at least 2 × 10^6^ CD34+/kg with an optimal target dose of 5–10 × 10^6^ CD34+/kg. Taking into account the HSPC loss due to cell manipulation (selection and transduction), a target range of 10–20 × 10^6^ CD34+ cells/kg at collection was considered for the BM harvest. A single BM harvest was sufficient for most of the patients to obtain the optimal target dose of haematopoietic progenitor cells for transplantation. Six out 57 patients were below the target range, but still above the minimal dose. For three of these an additional aliquot of purified CD34+ cells, previously collected during back-up leukapheresis harvest (*n* = 2) or BM (*n* = 1), was available and used to produce a second lot of transduced cells, in order to increase the amount of cells infused. Two additional patients received transduced cells obtained from the main BM harvest and previously stored CD34+ cells from BM (*n* = 1) or MPB (*n* = 1) (data not shown).

The BM procedure was overall well tolerated in all the children. No major complications were reported, despite the fact that the median volume collected was higher than the amount conventionally indicated and reported in previous published studies in paediatric healthy donors (up to 23.8 ml/kg) [18, 19] and in ADA-SCID patients who underwent GT in other centres (up to 20 ml/kg) [3, 15]. Moroever, no significant difference in CD34+ cell concentration was observed in patients who underwent a BM backup before the main GT BM harvest, suggesting that two collection procedures do not preclude harvesting of HSPCs for GT after a relatively short time interval (median of 23.5 days).

In a single-centre report on 109 paediatric healthy donors, BM volumes exceeded the standard BM volumes per body weight in 65% of the cases, with a reported median BM volume/kg of 18 ml, which was corresponding to half of our median values [21]. The authors did not report severe adverse events and suggested that larger volumes are safe, with the exception of the higher chances of being exposed to allogeneic blood products, which anyhow would be administered in the case of GT.

Another recent retrospective study showed large collected volumes harvested for the purpose of both autologous and allogeneic BM transplantations in children. Median amount of BM volume wascomparable with our results (37.66 ml/kg; range 20–55.3 ml/kg), with no mention about adverse events related to the procedures [22].

Concerning the harvest characterisation, a significant higher CD34+ percentage of cells was found in ADA-SCID BM compared with those of WAS and MLD patients. This difference can be ascribed to the know block in B-cell differentiation in the BM of these patients [23] and/or to the lower amount of nucleated cells intrinsically related to the immunodeficiency status of the disease. Indeed, a lower TNCcontent was documented in ADA-SCID patients compared with MLD patients. After normalisation for harvested volume and body weight, CD34+ cell content was similar in ADA-SCID patients with respect to WAS and MLD children. This indicates that the difference among patients was only relative to CD34+ cell proportion and not to absolute count.

The amount of BM CD34+ cell retrieved after harvest (median 0.5 × 10^6^/ml) was in line with the data from Furey et al., with a median of 0.7 × 10^6^/ml in children < 6 years [19], and higher than those reported in two other studies [22, 24].

There were minor discrepancies in the range of recoveries of CD34+ cells between the theoretical number at harvest, calculated based on interim analyses in the operating room and the final values counted at the GMP facility before starting the manipulation of the cells. These could be due to the different methods used for counting CD34+ cells. The cell yield after CD34+ cell purification (median 58.3%) was in line with a previous report [25] and should take into consideration the fact that the process also included a density gradient centrifugation in addition to immune-magnetic purification. Following ex vivo cell culture and transduction for a total of 3 days (MLD and WAS) and 4 days (ADA-SCID), the median yield of CD34+ cells was about 92.1%, with one-third of patients showing CD34+ cell expansion. A direct positive correlation was observed between selected and infused CD34+ cells per kilogram of body weight for all ADA-SCID, WAS and MLD groups, suggesting that, in spite of some variability among the patients, the steps leading to the final product could be considered stable and reproducible.

Donor/recipient characteristics are crucial factors that can influence stem cell yield and directly affect the outcome of gene therapy, which requires patients’ selected CD34+ cells as the starting product for gene modifications. The correlation between TNC and CD34+ cells collected and the clinical features of the children was evaluated, in order to establish if they could be direct indicators of rich or poor cell collection.

A significant difference between LI and EJ variants in MLD children was observed in terms of CD34+ cell content. This could be related to the fact that EJ MLD patients have a later onset and therefore are usually harvested at an older age, whereas only pre-symptomatic LI was enrolled in the MLD clinical trial. Indeed, CD34+ cell yield correlates inversely with weight and age at harvest in MLD children, while only a tendency was seen in ADA-SCID and WAS children.

Similarly to other reports, we found that significantly higher CD34+ cell counts are observed in younger donors [18, 19, 24]. Friebert et al. found that the cellularity of paediatric BM, including healthy donors, was the highest in patients younger than 2 years (~80%), declined to ~60% by the age of 5 years and then remained relatively constant in patients aged 5–18 years [26]. Furey et al. showed that the median CD34+ cell count obtained from paediatric sibling donors is higher in the younger donors, suggesting that the volume of the product harvested might be decreased without significantly changing the overall CD34+ cell count [19]. These observations were similar to the findings of this study in MLD patients, whose BM status may be considered closer to healthy donors, because of the absence of haematological and immunological defects. The same correlation was not documented in ADA-SCID and WAS diseases, where immunodeficiency is often associated with growth impairment and age increase does not always correspond to weight increase. Yet, a relationship between age of the donor and the dose of infused CD34+ cells after transduction was documented in ADA-SCID patients, similarly to the findings of Shaw et al. in their phase I/II trial of GT for ADA-SCID patients [15].

In summary, the cumulative data indicate that harvesting BM for the purpose of autologous gene therapy is a well-tolerated procedure that allows yielding an adequate stem cell amount for reaching a target cell dose at infusion of the engineered HSPCs. Additional studies are needed to compare the outcome of gene therapy after infusion of gene-corrected HSPC from different sources (BM and MPB).

## Supplementary information

Marked Supplementary data

Not marked supplementary data

Supplementary data
