# Supplementary material for: Bone marrow harvesting from paediatric patients undergoing haematopoietic stem cell gene therapy
Source: Bone Marrow Transplant. 2019 May 31;54(12):1995–2003. doi: 10.1038/s41409-019-0573-6 (PMC6897559; doi:10.1038/s41409-019-0573-6)
Supplement: Supplementary file 3 — Supplementary data [file 41409_2019_573_MOESM3_ESM.docx]

**SUPPLEMENTARY DATA**

The treatment was administered at the Paediatric Immunology and Bone Marrow Transplantation Unit at the San Raffaele Scientific Institute in Milan, Italy. All patients were evaluated as medically fit to donate by paediatricians and haematologists.

ADA-SCID patients were enrolled in a phase I/II clinical trial (NCT00598481) or treated under compassionate use (CUP). WAS patients were enrolled in a phase I/II clinical trial (NCT01515462). MLD patients were enrolled in a phase I/II clinical trial (NCT01560182) or treated with a hospital exemption program or CUP. Clinical information on 17 ADA-SCID, 3 WAS and 9 MLD patients have been previously reported [4, 5, 8]. Clinical information on the other patients will be reported elsewhere. Clinical trials and CUP were approved by the institutional ethics committee and Italian national regulatory authorities.

Processes of purification and transduction with gamma retroviral (ADA-SCID) or lentiviral vector (WAS and MLD) were previously reported [1, 5, 7].

All BM harvests were performed under general anaesthesia induced by fentanyl (1-2 µg/kg), propofol (2-3 mg/Kg) and rocuronium (0.5 mg/Kg) administration. BM morphology, karyotype and immunophenotype were investigated on a small initial aspirate before each patient underwent to BM back-up and main collection to exclude myelodysplasia and malignancies. BM collection was carried out via sequential single-hole 3 to 8 ml aspirations taken from the anterior and posterior iliac crests. Syringes were washed with a saline solution containing heparin at 200 IU/ml. Anticoagulant citrate dextrose was added to the collecting bag in the percentage of 10% with respect to the total BM harvested. BM was collected directly in 1.5 or 1.2 L collection bag, or using the BioAccess® Marrow Collection System kit or Fresenius Kabi® kit.

Being an autologous procedure in the context of an experimental product, the amount collected was higher than the one recommended for healthy paediatric allogeneic donor (10-20 ml/kg).

Indeed, BM volume collected was set at 20-30 ml/kg of donor weight according to the clinical trial protocols. The amount was adjusted for each patient consistently with the anesthesiologic evaluation and hemodynamic stability. In accordance to local standards, patients received supportive clinical care, fluid infusion, and albumin infusion, when required; patients were transfused with red blood cells to maintain haemoglobin at adequate levels. Procedures were well tolerated.

Three patients received BM transduced cells together with an aliquot of MPB CD34^+^ cells previously collected and cryopreserved during back-up; these cells were thawed and transduced in parallel with BM fresh cells in order to increase the dose of CD34^+^ cells/Kg. Similarly, 2 patients received fresh BM transduced cells together with an aliquot of thawed BM cells (collected previously, selected as CD34+ and cryopreserved). These HSPCs were infused together in order to increase the number of CD34+ cells/kg. One ADA-SCID patient received his back-up given the contamination of the major BM harvest during the first conditioning treatment. Later the patient underwent a second harvest from which both back-up and gene therapy CD34+ cells were collected [1].

To set the final volume and have an estimation of the final HSPC dose, we obtained data of TNC CD34+ cells during each BM harvest. We compared the estimated counts obtaining a similar trend from the one from the GMP facility (median estimated TNCx10^6^/ml: 11.4 in ADA-SCID *vs* 16.7 in MLD patients, p = <0.0001, Suppl. Fig 1A; median estimated TNCx10^8^/kg: 3.9 in ADA-SCID *vs* 6.1 in MLD patients, p <0.0001; Suppl. Figure 1B). First consecutive 15 ADA-SCID patients were not evaluated routinely for CD34+ cell count before manipulation.

The remaining ADA-SCID patients showed a higher amount of CD34+ cells compared to MLD patients. No significant difference was seen comparing estimated WAS CD34+% to MLD and to ADA-SCID (Suppl. Figure 1C). No significant difference in CD34+ percentage and cell concentration in the harvested BM was observed between male and female children for ADA-SCID and MLD patients.

During the pre-treatment phase, a back-up stem cells was foreseen for all the patients to be harvested and cryopreserved in case of poor engraftment or technical issues with product manufacture. The collection of the BM back-up (minimum dose 1x10^6^ CD34+ cells/Kg) was usually combined to central venous catheter positioning. Details on back-up harvests are reported in Suppl. Table 1.

In order to evaluate if interval between the 2 BM harvest (for back-up and for GT) was sufficient, we compared TNC and CD34+ cells for each pair of procedures. Focusing on TNCs concentration obtained in the 2 consecutive procedures, we observed that only within MLD patients’ TNC were higher at back-up harvest (TNC 20.7 x10^6^/ml at back-up vs 18.2 at GT; paired t test, p 0.04) (Suppl. figure 2A). A cumulative comparison between TNC/ml for back-up and for GT for all the patients showed a statistically significant difference (Suppl. figure 2B), probably due to the higher number of MLD patients (paired t test, p 0.03). On the contrary, no significant difference was observed between the CD34+ cell concentrations determined during back-up collection and harvest for GT (Suppl. figure 3A), suggesting that the HSPCs pool is re-established after the first BM collection. In order to determine if the applied minimum time interval between back-up harvest and harvest for GT was adequate to allow recovery of the BM and permit an adequate yield for GT, a further analysis was performed to investigate the relation between the difference in HSPCs amount at back-up and GT and the days between the two procedures (median 23.5 days, range 7-363 days) in the total group including all patients analysed (n=42). In patients who underwent 2 BM collection in less than 23.5 days of interval (n=27), CD34+ cell concentration remained overall stable (CD34+ 0.68 vs 0.61 x10^6^/ml, p 0.23) (Suppl. figure 3B).

**Supplementary figures:**

 **Suppl. Figure 1:** Data from 18 ADA-SCID, 5 WAS and 25 MLD patients are available for estimated TNC (A-B). Data from 8 ADA-SCID, 5 WAS and 28 MLD patients are available for estimated CD34+ cells’ percentages (C).

**Suppl. figure 2:** TNC concentration obtained in the 2 consecutive BM harvest for back-up and GT purpose in MLD (A) and all the patients (B).

**Suppl. figure 3:** CD34+ cell concentration in the 2 consecutive BM harvest for back-up and GT purpose in all the patients (A) and in patients who underwent 2 BM collection in less than 23.5 days of interval (B).

**Supplementary table:**

| **Disease** | **Sex (M/F)** | **Weight (Kg)** | **Harvested Volume (ml)** | **Vol/Kg (ml)** | **TNC/Kg**  **(x10^8^/Kg)** | **CD34+ (%)** | **CD34+/Kg (10^6^/Kg)** | **Cryopreserved CD34+/Kg (10^6^/Kg)** |
| --- | --- | --- | --- | --- | --- | --- | --- | --- |
| **ADA-SCID**  **(n=22)** | 16/6 | 9.6  (4.5-26.0) | 40.0  (22.0-161.0) | 3.8  (1.8-12.9) | 0.6  (0.2-1.9) | 6.5  (2.0-8.8)* | 6.3  (1.2-8.3)* | 2.5  (0.1-7.9) |
| **WAS**  **(n=5)** | 5/0 | 11.5  (7.8-13.2) | 67.1  (48.2-114.7) | 7.0  (4.5-9.2) | 1.7  (0.6-2.4) | 3.6  (1.9 - 5.6) | 6.0  (1.6 - 9.4) | 5.5  (2.0-7.6) |
| **MLD**  **(n=29)** | 16/13 | 10.3  (7.0-32.0) | 120.0  (42.7-918.0) | 11.6  (4.9-36.7) | 2.5  (0.7-7.7) | 2.9  (1.1 - 5.7) | 7.4  (2.0 - 14.7) | 4.3  (1.3-12.7) |
| **Total**  **(n=56)** | 37/19 | 10.3  (4.5-32.0) | 74.0  (22.0-918.0) | 7.7  (1.8-36.7) | 1.5  (0.2-7.7) | 3.3  (1.1-8.8) | 6.6  (1.2-14.7) | 4.2  (0.1-12.7) |

**Suppl. table 1:** Cell harvest parameters from BM back-up (median and range reported) for ADA-SCID, WAS and MLD patients.

*Data available for the last 10 consecutive ADA-SCID patients
